# Addressing GI Health Through the Bidirectional Modulation of the Gut-Brain Axis With Herbal Extracts: A Narrative Review

**DOI:** 10.7759/cureus.66698

**Published:** 2024-08-12

**Authors:** Cassandra Evans, Douglas Kalman

**Affiliations:** 1 Health and Human Performance, Nova Southeastern University, Davie, USA; 2 Human and Sport Performance, Rocky Mountain University of Health Professions, Provo, USA; 3 Nutrition, Nova Southeastern University Dr. Kiran C. Patel College of Osteopathic Medicine, Davie, USA; 4 Research Division, Substantiation Sciences, Inc., Weston, USA

**Keywords:** gut-brain connection, gut health, functional constipation, functional gastrointestinal disorder, gut-brain axis

## Abstract

Functional gastrointestinal disorders (FGIDs) refer to a group of disorders with chronic symptoms, such as abdominal pain, dysphagia, dyspepsia, diarrhea, constipation, and bloating. Among these, functional constipation significantly impacts the quality of life and is linked with comorbidities, such as anxiety and depression. The exact pathophysiology remains unclear despite the widespread occurrence. Research suggests that the gut-brain axis plays a role in FGIDs. Disruptions in the bidirectional communication between the brain and gastrointestinal (GI) tract contribute to GI symptoms and mood disturbances. The incomplete understanding of FGID pathophysiology has led to limited treatment options. Traditional treatments often focus on single symptoms and come with side effects, prompting the need for alternative approaches that address both GI and psychological components. Alternative approaches including herbal supplements offer a natural alternative to conventional medicine by promoting regularity and gut health. *Abelmoschus esculentus* L. or okra has a history of use in traditional medicine. Bioactive compounds such as polysaccharides and fibers found in okra offer gastroprotective benefits. *Withania somnifera* is a plant commonly referred to as ashwagandha. The plant root has been used for its health-promoting effects. Research supports the use of *W. somnifera* to help with stress and sleep. Digexin is a herbal supplement combining *W. somnifera *(ashwagandha) and *A. esculentus* (okra). It has shown promise in improving both GI regularity and mood by modulating the gut-brain axis. Clinical studies support the potential of a novel herbal supplement that aids in the management of FGIDs. This narrative review looks at FGIDs, etiologies, current treatment, and possible therapeutic supplements to aid in symptom management.

## Introduction and background

Functional gastrointestinal disorders (FGIDs) impact 40% of the global population [1-3]. FGIDs are a group of disorders with no identified etiology characterized by chronic gastrointestinal (GI) symptoms, including abdominal pain, dysphagia, dyspepsia, diarrhea, constipation, and bloating [2,3]. FGIDs are differentiated into 33 different disorders in adults, with altered bowel habits being one of the most common [3,4]. Of people meeting the criteria for FGIDs, two-thirds seek medical help yearly, 40% use regular medication, and one-third undergo potentially unnecessary abdominal surgery [2,3]. FGIDs, including constipation, are associated with several comorbidities, including anxiety and depression [5]. Functional constipation or unsatisfactory defecation and difficult or infrequent stools affect 9-20% of the US population [6]. It is estimated to impede an individual’s overall productivity and interfere with daily quality of life [6]. Treatment, usually osmotic laxatives, focuses on one symptom and is frequently accompanied by unpleasant side effects [7]. Alterations in mood and mental health frequently occur with FGIDs [2,3]. These conditions were traditionally thought to have no explainable origin [2,3]. However, recent clinical approaches consider that these conditions arise due to alterations in the gut-brain axis, which helps to explain why they are often associated with disruptions in other physiological systems and are commonly associated with mood disturbances and decreased quality of life [1,2]. Multidisciplinary clinical care involving gastroenterologists, nurses, dietitians, and psychologists seems to be more successful than gastroenterologist-only care when it comes to improving symptoms, psychological well-being, quality of life, and treatment costs for FGIDs [3]. In addition, considerations for alternative, more natural options that address gut function and brain function are needed. One such option is Digexin, a herbal combination of *Withania somnifera* (ashwagandha) and *Abelmoschus esculentus *(okra), which improves regularity and supports mood by mediating the gut-brain axis. 

FGIDs

FGIDs refer to a group of disorders contributing to chronic GI discomfort. The most common and consequently the most studied FGIDs are irritable bowel syndrome (IBS), functional constipation (FC), functional diarrhea, and functional bloating/distention [1]. Due to the absence of structural or biochemical abnormalities, FGIDs are primarily symptom-based [3,8]. They are classified by a cluster of symptoms diagnosed according to the Rome criteria [4]. Symptoms range from mild to severe, all of which interfere with daily living or cause emotional distress to those affected. FGIDs are correlated with a decreased quality of life, and those suffering from concurrent or overlapping symptoms experience an even greater reduction in quality of life [9]. While traditionally FGIDs were viewed as something with no organic basis, recently, the view has shifted to an understanding that symptoms stem from a disruption in the gut-brain axis [2,4]. 

GI tract symptoms include abdominal pain, diarrhea, constipation, bloating, fullness, nausea, and vomiting [2]. Societal views and values influence how symptoms are perceived as a sign of illness. Symptom perception may be considered problematic in one group but marginalized in another due to prevalence, culture, lifestyle/dietary differences, and genetics [2,4]. Symptom overlapping is common, with multiple FGIDs or other unexplained disorders like chronic fatigue syndrome or fibromyalgia often coexisting [2,3]. Impaired sleep, anxiety, and depression prevalence rise with the number and severity of FGIDs [2]. A dynamic relationship exists between early life experiences, learned behaviors, psychological distress, coping strategies, and changes in GI physiology [3]. Therefore, management includes managing behaviors, thoughts, and beliefs in addition to abnormal physiology [3].

Constipation is a GI dysfunction that affects an average of 15% of the global population [7,10] and is the most common bowel disorder [1]. Individuals with chronic constipation are more likely to experience comorbidities compared to other GI disorders [9]. Comorbid constipation is common in people with poor sleep quality, increased stress, anxiety, and depression [7]. Kaplan et al. reported high rates of functional dyspepsia and functional anorectal pain in individuals with disturbed moods. The authors suggest that this is related to colonic distension from fecal loading. Constipation accompanied by pain and discomfort is positively correlated with higher levels of depression and poorer quality of life [9]. Uncertainty over when symptoms will present themselves or get worse and persistent symptoms contribute to anxiety and the burden imposed by functional constipation [11]. Medications such as stool softeners, laxatives, or smooth muscle stimulants and dietary and behavioral modification are typically initial approaches to managing constipation [7].

Etiology

The cause of FGIDs is multidimensional and involves alterations in GI motility, visceral hypersensitivity, the interplay between the bidirectional dysregulation of the gut-brain interaction (the gut-brain axis), altered central nervous system (CNS) processing of sensory input, psychosocial disorders, altered microbiota, increased intestinal permeability, and low-grade infiltration [4,12].

Alterations in GI motility such as muscular spasms lead to pain or abdominal discomfort. A slow rate of motility leads to constipation or, conversely, hypermotility resulting in diarrhea [13]. The different mechanisms or combinations of mechanisms contribute to the large variation in symptoms reported.

Often called the gut’s mini-brain, the enteric nervous system (ENS) is a complex network of neurons found in smooth muscle cells throughout the GI tract. The ENS regulates GI functions including motility. The ENS affects immune and endocrine cells, which indirectly influence the GI function [14,15]. Although the ENS can function on its own, it receives input from the CNS via the sympathetic and parasympathetic pathways [14]. This interaction is commonly referred to as the gut-brain axis. Disrupted or impaired communication between the gut and the brain is associated with increased sensitivity to pain and altered GI function. Moreover, psychological disturbances such as anxiety and depression are common in FGIDs. Research suggests that psychological disturbances precede GI symptoms in half of FGID cases; the other half of cases start with GI symptoms, followed by psychological disturbances [2,16]. Life stressors, disrupted sleep patterns, and traumatic life experiences impact individuals on a physiological and psychological level. Psychosocial disturbances have profound effects on the gut, contributing to the development and exacerbation of GI disorders. Stress increases gut permeability and alters gut microbiota. Disruptions in gut permeability are correlated with a pro-inflammatory state; however, it is unclear which occurs first [17]. Perturbations in the gut microbiome are correlated with diseases that extend beyond the GI tract. Dysbiosis or imbalance of the bacteria in the gut microbiome can alter gut function, specifically motility [15]. Infections such as *Helicobacter pylori* lead to changes in the gut microbiome that can persist after the infection is cleared [2,16]. Following an infection, acute inflammation signals the body’s healing process. However, low-grade inflammation affects neuron function and the release of cytokines and neuropeptides. These alterations change gut function and increase intestinal immune activation leading to a variety of symptoms such as visceral hypersensitivity and delayed gastric emptying [2,16].

Constipation is characterized by infrequent bowel movements or difficult passage of stools, often accompanied by straining, abdominal discomfort, and a sense of incomplete evacuation. Constipation can occur from a multitude of different factors [18,19]. Nutritionally, inadequate water intake leads to hard or lumpy stools while low fiber intake makes stools smaller and harder to pass. Certain medications and structural and muscular abnormalities slow transit time [19]. Similar to acute constipation, symptoms of functional constipation include straining or difficulty defecating, lumpy or hard stools, sensation of incomplete evacuation, or anorectal blockage [20]. Functional constipation is characterized by experiencing two or more symptoms for three months or longer [20]. Structural abnormalities are not present in functional constipation. Rather faulty communication between the gut-brain axis, alterations in GI motility, visceral hypersensitivity, altered microbiota, increased intestinal permeability, low-grade infiltration, and altered CNS processing of sensory input are thought to contribute to functional constipation [2,4].

Unfortunately, treatment is often focused on one condition such as constipation, or a disturbance in mood, rather than considering the interrelationship of the issues. Quality of life may be drastically reduced in FGIDs due to symptoms such as constipation [21]. Constipation in this condition creates GI discomfort that may impact physical well-being. Thus, the treatment often focuses on relief of symptoms [21]. Generally, prescription medications should complement dietary or lifestyle changes for mild chronic symptoms and be used primarily during acute exacerbations [4]. However, the use of pharmacological agents has led to increases in consumer dissatisfaction related to unwanted side effects and insufficient and erratic results [7]. Bascilisco et al. evaluated 81 outpatients (33 with IBD with constipation and 48 with chronic constipation) and found that 43% did not feel they received adequate relief from their symptoms. The patients with more severe symptoms and lower BMI were more likely to have unfavorable results. Over-the-counter (OTC) medications such as laxatives, stool softeners, fibers, prebiotics, and probiotics are often used to manage constipation [22].

In the USA, more than 1.5 billion dollars were spent on laxatives in 2019 [7]. Laxatives can improve stool frequency but can also make bloating worse; therefore, they often need to be coupled with another medication to manage other symptoms [3]. Laxatives may successfully improve bowel function but are associated with side effects when used long term and do not address the co-related conditions. Emerging evidence suggests that laxatives may cause changes to the gut microbiota and are associated with higher rates of dementia [23-25]. More research is needed to better understand the type and duration of effects caused by laxatives. Vriesman et al. reported that more than half of adults with functional constipation were dissatisfied with treatment due to a lack of efficacy and adverse events [20]. As a result, the demand for alternative methods to minimize discomfort associated with constipation and enhance GI function is increasing [7]. Considering the multiple comorbidities and complex nature of the condition, it is unlikely that a single treatment or supplement will help all individuals suffering from constipation related to FGIDs.

Over time, the understanding of FGIDs has evolved from a simplistic view to a more comprehensive bio-psychosocial model. Initially, examinations of psychosocial aspects of disease were not explored. In the late 1980s, the medical community started to explore the psychosocial aspects of GI disorders [4]. It is now characterized by gut-brain interactions that encompass a range of disorders marked by GI symptoms associated with motility dysfunction, altered gut microbiota, altered immune and mucosal function, visceral hypersensitivity, and CNS processing. The brain and gut, unlike other organs, share a linked nervous system derived from the embryonic neural crest. This also explains why emotional factors such as stress and anxiety are closely linked to the overall gut function [4]. Thus, current advances in treatment should focus on multi-ingredient supplements that address the co-existing conditions of GI health along with alterations in stress, sleep, mood, and feelings of anxiety.

## Review

Gut-brain axis

The gut-brain axis refers to the bidirectional communication network between the CNS and the ENS [13,26]. This complex communication has a direct effect on gut functioning and is thought to affect cognitive function and mood. This intricate communication network utilizes different neural, hormonal, and immunological signaling pathways to facilitate constant communication between the gut and the brain The hypothalamic-pituitary-adrenal (HPA) axis, which is linked to the autonomic nervous system (ANS) and nerves in the GI tract, facilitates the body’s adaptive response to stress [27]. This allows the brain to influence the GI tract via functional immune effector cells and enteric neurons [28]. The gut microbiota, comprised of trillions of microorganisms, directly influences gut health and function. Numerous bioactive compounds are produced by the gut microbiota including short-chain fatty acids (SCFAs) and neurotransmitters. This influences brain neurochemistry and in turn affects mood and cognitive function. Research involving germ-free animals demonstrates alterations in metabolic function, abnormalities in the CNS, and impaired gut function. Acute stressors disrupt the production and release of gastric secretions and the mucous layer of the GI tract [27]. Emotions like fear, anger, anxiety, painful stimuli, and physical stress cause disruptions in the intestinal transit [4]. Sympathetic activation within the gut-brain axis results in gastroparesis and reduced gastric acid secretion, which then contributes to GI dysfunction, including constipation [7]. Decreased gastric motility and mood dysfunction are associated with decreased serotonin levels resulting in changes in bacteria growth within the GI tract [7]. Liang et al. [29]. reported a positive correlation between mood disturbances (anxiety and depression) and gut microbiota in Chinese women with functional constipation. Strategies to manipulate bacteria in the gut, such as probiotics, decrease depression and anxiety-related behaviors [28].

The gut-brain axis has profound effects on both physical and mental health. Dysregulation in bidirectional communication is linked to the pathogenesis of numerous GI disorders, including FGIDs [30]. Dysregulation in the gut-brain axis contributes to the development and ongoing symptoms associated with FGIDs and alterations in gut microbiota composition and diversity [27,30]. A retrospective study conducted by Jones et al. [31] reported that twice as many patients were diagnosed with mood or anxiety disorder prior to FGIDs compared to those diagnosed with FGIDs first. The authors noticed experiencing multiple FGID symptoms, which affect the quality of life, may exacerbate psychological symptoms. As mentioned previously, psychosocial stressors modulate gut function through the activation of the HPA axis and the ANS, leading to changes in gut motility, visceral sensitivity, and immune responses while changes in gut microbiota can disrupt intestinal barrier function, increase gut permeability, and trigger immune activation and low-grade inflammation [27,28]. While not diagnostic prerequisites, psychosocial factors influence GI tract functioning via the brain-gut axis (motility, sensitivity, and barrier function), shape pain experience and symptom behavior, and impact treatment choices and clinical outcomes [4]. Awareness of this bidirectional communication has changed how GI issues are managed and assessed [2].

Alternative management

As discussed, the multiple symptoms of functional constipation can significantly impact the quality of life in many dimensions of health, including social, emotional, and physiological [1-3]. However, prescription medications or OTCs may only address the constipation treatment, fail to address the multi-dimensional complex elements contributing to impaired gastric function, and are most likely not suitable for long-term use [7]. Nutraceuticals are dietary supplements that contain vitamins, minerals, herbs, or other extracts that provide health benefits or have therapeutic effects. When used as a part of a holistic approach to health, nutraceuticals may offer additional benefits and less long-term side effects and may complement conventional medical treatment [32]. There are numerous nutraceuticals or herbal extracts that aid in the prevention or relief of constipation including senna, cascara, and aloe [18].

Although less studied as a nutraceutical, okra (*Abelmoschus esculentus* L.), commonly known as lady’s fingers, possesses many health benefits as a food. Okra pods contain several bioactive phytochemicals and are rich in vitamins, minerals, carbohydrates, dietary fiber, and unsaturated fatty acids such as linolenic acid and oleic acid [7]. Consuming the pod improves the nervous system, brain, heart, stomach, muscle, and intestinal function. It has traditionally been used for gastroprotective, antiulcer, and diuretic benefits [33]. Yasin et al. reported the protective effects of okra extract and normal gastric mucosa in rats with aspirin‐induced gastric ulcers [34]. The gastro-protective effects are related to the rhamnogalacturonan polysaccharide and glycosylated compounds found in okra fruits. These work by inhibiting *H. pylori* adhesion to the gastric mucosa [7]. Okra contains flavonoids such as quercetin and other polyphenols, which exert anti-inflammatory and antioxidant properties [7,33]. The antioxidant and anti-inflammatory activities may help explain many of okra’s health benefits, including its neuroprotective effects [35].

*Withania somnifera* (L.) dunal (Solanaceae), also called “Indian winter cherry” or “Indian ginseng,” is traditionally used for stress, sleep, and constipation [5,7,36]. Extracts of *W. somnifera* have demonstrated neuroprotective and psychoactive activities in preclinical and human studies [36]. Withanolides (steroidal lactones), flavonoids, phenolic acids, steroidal alkaloids, saponins, and tannins are the major pharmacologically active phytochemicals found in the fruit of *W. somnifera*. The withanolides combined with other bioactive compounds in *W. somnifera* reduce the sympathetic tone of the CNS. This leads to enhanced gastrin secretion that improves peristalsis. In addition, this impact on the CNS improves sleep and stress and decreases depression [7]. Research suggests that *W. somnifera *modulates neurotransmitters such as gamma-aminobutyric acid (GABA) and serotonin [37-41]. Some animal studies have demonstrated that *W. somnifera* extracts can enhance GABAergic signaling by increasing GABA receptor activity or GABA release [39,40]. *W. somnifera*‘s ability to modulate GABAergic neurotransmission may be responsible for its anxiolytic effects. In addition, *W. somnifera*’s effects on the ANS may further enhance its anxiolytic effects and promote relaxation leading to better sleep quality and duration [37]. Serotonin is involved in a wide range of physiological processes [42]. Serotonin is more commonly recognized for its role in brain function, specifically mood regulation and sleep-wake cycle. Serotonin also exerts its effects on the body. It regulates GI functions, including peristalsis and secretion of digestive juices. Studies have suggested that *W. somnifera*'s effects extend beyond mood and can include gut health [37,41,43,44]. One of the significant components in the extract is glycowithanolides, which are primarily responsible for the neuroprotective effect. Glycowithanolides possess superior lipid peroxidation inhibition abilities. The antioxidant properties found in this herb are comparable to curcumin and ascorbic acid [36]. *W. somnifera* is an adaptogen aiding in the body’s ability to alleviate stress, anxiety, and fatigue while enhancing overall well-being [7]. In animal models, oral supplementation can reduce proinflammatory cytokines, reactive oxygen species (ROS), tumor necrosis factor (TNF), and nitric oxide (NO) in animal models [36]. Human clinical studies report reductions in serum cortisol levels, stress, and anxiety [7]. Daily supplementation of 240 mg was associated with decreased cortisol and dehydroepiandrosterone levels in the morning and decreased anxiety and stress. In a double-blind, randomized, placebo-controlled trial, a high-concentration full-spectrum extract of *W. somnifera *root decreased serum cortisol levels and improved resistance to stress. Pretreatment of *W. somnifera* in rats deprived of sleep for 12 hours resulted in the reversion of sleep-deprived neuromotor dysfunction and memory loss along with improvement in alertness, exhortative nature, and memory [36]. These studies demonstrate the potential benefits of ashwagandha for brain health.

Digexin

**Figure 1:**
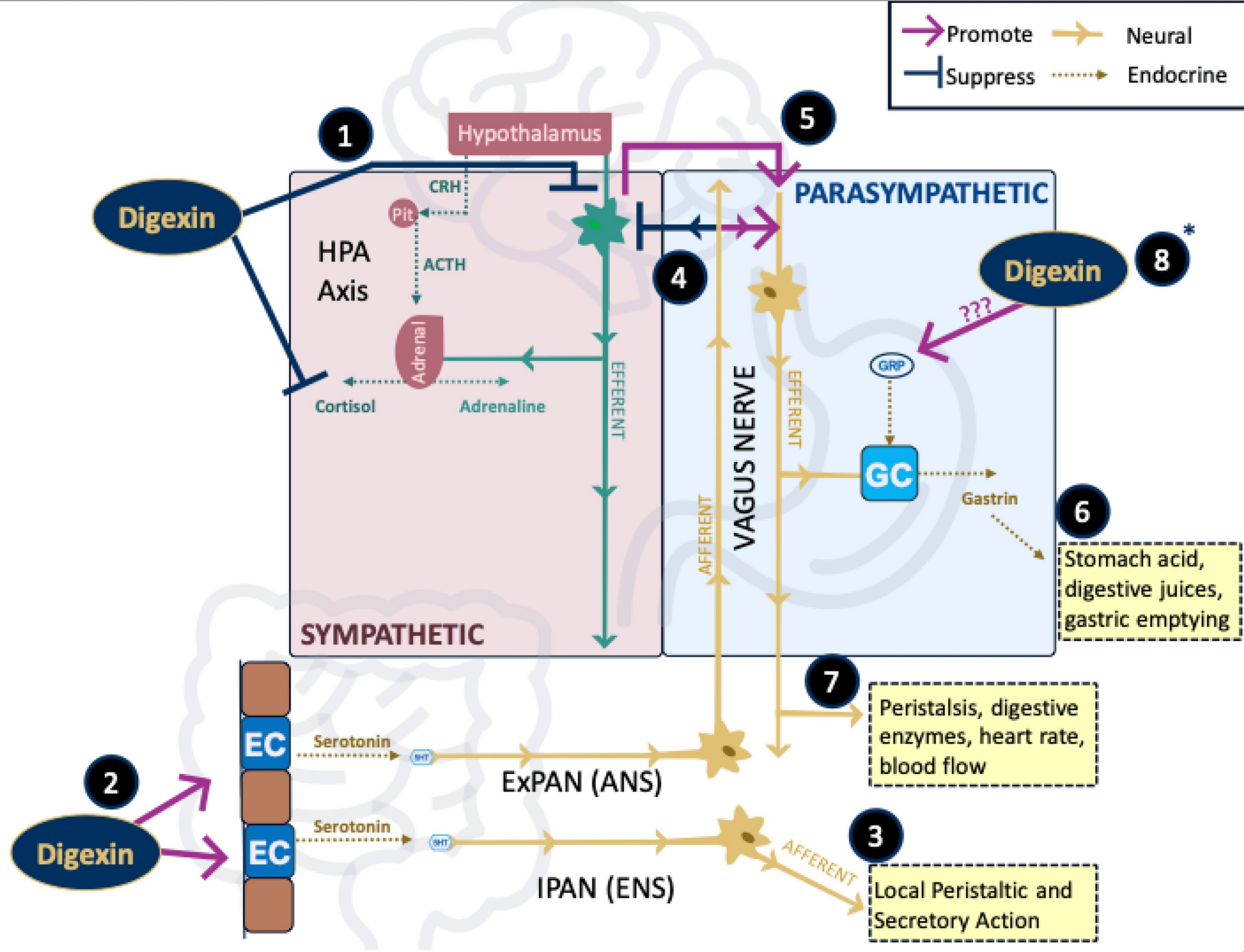
Digexin's mechanisms of action 1) Digexin reduces stress and lowers cortisol. The precise mechanism is still unknown, but it has the effect of suppressing the sympathetic nervous system (SNS). 2) Digexin boosts serotonin levels, likely through stimulation of enterochromaffin (EC) cells in the gut lining. 3) Increased serotonin has the immediate effect of stimulating the enteric nervous system (ENS) through intrinsic primary afferent neurons (IPANs) and activating local peristalsis/secretions. 4) Serotonin signals through vagal extrinsic primary afferent neurons (ExPANs), which suppress the SNS and promote the peripheral nervous system (PNS). 5) PNS is highly preferred now due to suppressed SNS from decreased stress and ExPAN promotion of PNS. 6) PNS stimulates G cells to produce gastrin, which produces stomach acid, digestive juices, gastric emptying, and peristalsis. 7) The PNS tone is conducive to digestion, stimulating peristalsis, and releasing enzymes and hormones, along with other factors. 8. *Potential secondary activation of gastrin Digexin through an increase in gastrin-releasing peptide (theoretical). This figure was created by Eric Withee and Doug Kalman.

Digexin is a combination of *W. somnifera *(ashwagandha) and *A. esculentus* (okra), which have been shown to work synergistically to stimulate peristalsis and improve barrier function. This synergetic combination has demonstrated adaptogenic function and has been shown to increase serotonin [7]. Unpublished, preclinical studies reported enhanced effects on GI contractility in a multi-ingredient supplement compared to single herbal extracts. Based on the known benefits of Digexin’s key ingredients, this supplement is designed to address both physiological and psychological elements of functional constipation (Figure 1).

In a proof-of-concept, randomized, double-blind, placebo-controlled study, 48 adult men and women with functional constipation received either 300 mg or 500 mg of a botanical blend or placebo for 14 days. Participants receiving both doses of the supplement experienced a significant reduction in patient-reported constipation symptoms, improved quality of life, improvement on the Gastrointestinal Symptom Rating Scale (GSRS) scores, improved sleep quality, and reduced stress. At the conclusion of the study, the participants exhibited remarkable reductions in interleukin-6 and cortisol levels in addition to rises in serum serotonin, gastrin, and interleukin-10 levels. There were no reported adverse events. The limitations of the study were the size (this was only a proof-of-concept study) and that participants followed their usual diets [7]. A similar study of 135 men and women receiving the same doses for 60 days revealed that participants consuming both doses experienced a significant reduction in constipation and related symptoms, GI symptoms, improved quality of life, bowel performance, anxiety, depression, and sleep quality. They experienced a significant improvement in serotonin, gastrin, diamine oxidase (DAO), and anti-inflammatory cytokines and decreased levels of serum cortisol, zonulin, and pro-inflammatory IL-6. Improvements in serotonin and gastrin indicate enhanced secretion of gastric mucus and peristalsis. There were no serious side effects [45]. The large effect sizes and significant improvements in outcomes demonstrate Digexin’s direct benefits on mood and GI function via the gut-brain axis.

Reductions in cortisol can lead to improved GI function. Lower cortisol levels are associated with decreased sympathetic activity and increased parasympathetic activity [46]. This shift increases blood flow to the GI tract, stimulates digestive enzyme secretion, and enhances GI motility [47]. Serotonin is a neurotransmitter primarily known for its role in regulating mood and emotions in the brain. Serotonin transmits signals through the vagus nerve, which influences emotional and cognitive processes [48]. Increases in serotonin improve mood, sleep, and anxiety. *W. somnifera *supplementation alleviates stress and anxiety and improves the quality of life, which is partly related to increases in serotonin [41]. Serotonin influences parasympathetic tone indirectly through its effects on the ENS [48]. This stimulates peristalsis, which aids in digestion and nutrient absorption. Gastrin promotes gastric emptying, leading to a faster transit of food from the stomach into the small intestine [49]. Increases in gastrin such as those observed in individuals taking Digexin indicate that it benefits the entire digestive tract. The aforementioned studies combined with the effects and research surrounding its key ingredients suggest that Digexin has multiple benefits for GI function, brain function, and communication between the two.

## Conclusions

FGIDs are a common occurrence globally. Functional constipation is one of the most common FGIDs. Decreased gastric motility and mood dysfunction commonly occur concurrently in FGIDs, especially functional constipation. Research now recognizes that FGIDs are related to the gut-brain axis. Disrupted communication within the gut-brain axis results in gastroparesis and reduced gastric acid secretion, which then contributes to GI dysfunction, including constipation. Awareness of this bidirectional communication has changed how GI issues are assessed and managed. Current treatments often target a single symptom and fail to address the mental-emotional component. Digexin, a herbal combination of *W. somnifera* (ashwagandha) and *A. esculentus* (okra), enhances GI function and supports wellness. This combination of herbs shows promise with significantly fewer adverse reactions to traditional treatment while being able to address the brain-gut axis. *W. somnifera *(ashwagandha) and *A. esculentus *(okra) are cost-effective natural sources of vitamins, minerals, carbohydrates, dietary fiber, and other bioactive compounds that are important for well-being. This review explored the potential benefits of combined ingredients, known as Digexin, on GI function and well-being. Future studies should explore the impact of these combined botanicals on health and well-being.
